# Effects of Graphene Oxidation on Interaction Energy and Interfacial Thermal Conductivity of Polymer Nanocomposite: A Molecular Dynamics Approach

**DOI:** 10.3390/nano11071709

**Published:** 2021-06-29

**Authors:** Francesco Maria Bellussi, Carlos Sáenz Ezquerro, Manuel Laspalas, Agustín Chiminelli

**Affiliations:** Aragon Institute of Technology ITAINNOVA, 50018 Zaragoza, Spain; francesco.bellussi@polito.it (F.M.B.); mlaspalas@itainnova.es (M.L.); achiminelli@itainnova.es (A.C.)

**Keywords:** polymer nanocomposite, graphene, interfacial properties, interaction energy, kapitza resistance, molecular dynamics

## Abstract

Interfacial characteristics of polymer nanocomposites represent a crucial aspect to understand their global properties and to evaluate the interaction between nanofillers and matrix. In this work we used a molecular dynamics (MD) approach to characterize the interfacial region at the atomistic scale of graphene-based polymer nanocomposites. Three different polymer matrixes were considered, polylactic acid (PLA), polypropylene (PP) and epoxy resin (EPO), which were reinforced with three types of graphene fillers: pristine graphene (G), graphene oxide (GO) and reduced graphene oxide (rGO). In particular, the compatibility of the nanofillers in polymer matrixes were evaluated in terms of the interaction energy, while the interfacial thermal resistance (Kapitza resistance) between matrices and fillers was calculated with a nonequilibrium molecular dynamics (NEMD) method. Results showed that the oxidation degree plays an important role on the studied properties of the interfacial region. In particular, it was observed that the Kapitza resistance is decreased in the oxidized graphene (GO and rGO), while interaction energy depended on the polarity of the polymer matrix molecules and the contribution of the Coulombic component.

## 1. Introduction

In the last decade, graphene (G) has attracted the attention of both scientists and industry stakeholders for its extraordinary mechanical, electrical and thermal properties [1,2,3,4], making it one of the best candidates for the development of nanomaterials in a wide range of applications [3,5,6]. Surface modified derivatives of graphene have been proposed, such as graphene oxide (GO) and reduced graphene oxide (rGO), with the aim of increasing the compatibility with polar matrices [7,8] and improving the composite interface characteristics through surface functionalization [9,10].

Molecular dynamics (MD) simulations have given light to the study and knowledge of the interfacial properties between matrix and filler, which represent key aspects to evaluate the dispersibility and binding capacity of nanofillers in polymer matrix and to understand the effective global characteristic of a nanocomposite. Regarding thermal properties, for example, Su et al. [11] developed a new effective-medium theory introducing both the thermal boundary resistance between matrix and filler (Kapitza resistance, R_k_) and graphene–graphene contact resistance. By comparing analytical and experimental results these authors demonstrated that the interfacial thermal properties play a relevant role in the global thermal conductivity of a nanocomposite. Similarly, Bigdeli and Fasano [12] and Mohammad Nejad et al. [13] demonstrated, through reverse nonequilibrium molecular dynamics (RNEMD) simulations, that both geometry properties and the thermal boundary resistance between fillers affect significantly the overall thermal properties. In terms of the interactions between the matrices and the fillers, Sáenz et al. [14] evaluated the interface thickness of composites based on epoxy resin and triple wall carbon nanotubes (TWCNT), by analyzing the density profile. Sun et al. [15] calculated the interaction energy between epoxy resin and GO depending on the content of hydroxyl groups, identifying the range in which the interfacial energy seemed to decrease more sensibly by increasing the number of hydroxyls. MD studies also allow the analysis of the effects of filler surface chemical modification on the interfacial properties. He et al. [16] and Mortazavi et al. [17] found that functionalization and the creation of covalent bonds between matrix and filler reduces sensibly the thermal boundary resistance, thus improving thermal conductivity. 

In this study, molecular models of nanocomposites based on different polymer matrices (poly lactic acid, polypropylene and epoxy resin) and graphene fillers (pristine and oxidized) were developed, with the aim of studying some of their interfacial properties. The interface energy between matrix and filler was first evaluated to obtain an estimation of the compatibility, and then the interfacial thermal resistance across the interface (Kapitza resistance) was calculated to quantify the effect of the oxidation degree on interfacial thermal transport. 

## 2. Materials and Methods

**Figure 1:**
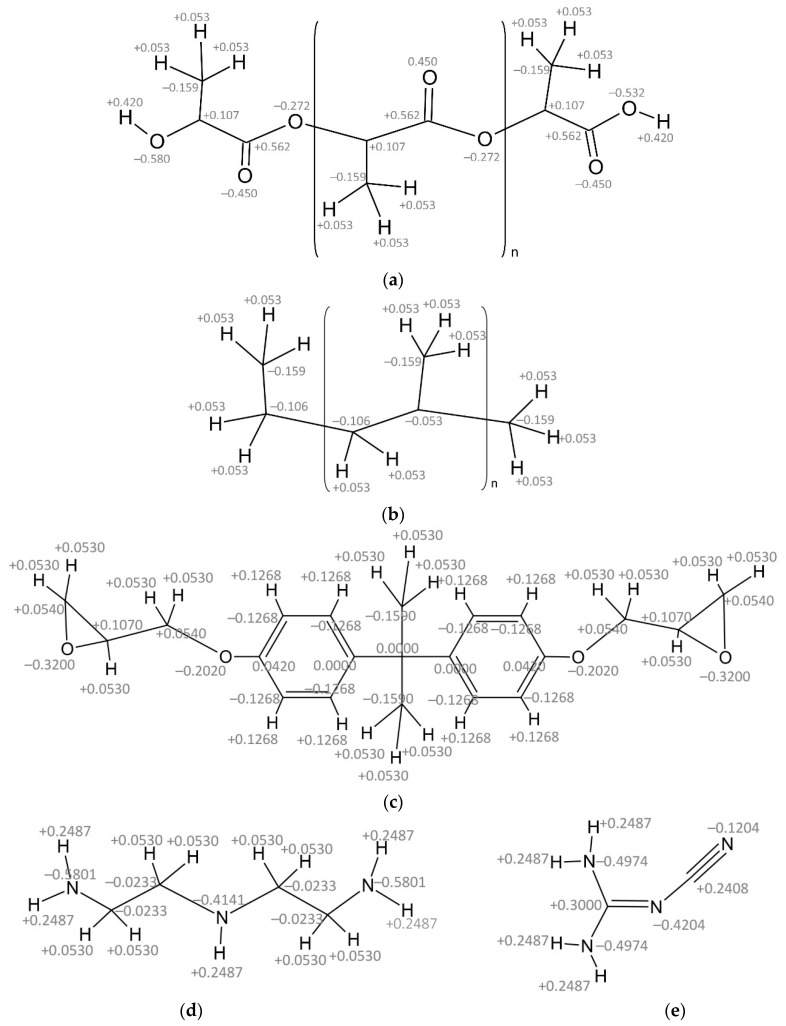
Structure and partial atomic charges of polymers: (**a**) polylactic acid (PLA); (**b**) polypropylene (PP); epoxy resin molecules: (**c**) DGEBA, (**d**) DETA and (**e**) DICY.

Three types of nanocomposites were modeled with atomistic detail by considering three different polymer matrices: two thermoplastic polymers, Polylactic acid (PLA) and Polypropylene (PP), and a variety of an epoxy thermoset resin (EPO). The chemical structure of generic PLA and PP chains are shown in Figure 1a,b, where the partial atomic charges are indicated. In all the cases studied, the isotactic variety of the polymers (i.e., with the methyl groups in the same side of the chain in the full extended conformation) was considered. The epoxy resin (EPO) is based on diglycidyl ether of bisphenol-A (DGEBA) with dicyandiamide (DICY) and diethylene triamine (DETA) as hardeners. The chemical structure of DGEBA, DICY and DETA with the partial charge is shown in Figure 1c–e. The COMPASS force field [18] was used to model the interactions between the atoms belonging to the polymer chains. The energy potentials stated in this force field are given in Equation (1).

(1) $$\begin{array}{l} E_{pot}=\underset{b}{\sum }\left[K_{2}{\left(r-r_{0}\right)}^{2}+K_{3}{\left(r-r_{0}\right)}^{3}+K_{4}{\left(r-r_{0}\right)}^{4}\right]\\ +\underset{\theta }{\sum }\left[H_{2}{\left(\theta -\theta_{0}\right)}^{2}+H_{3}{\left(\theta -\theta_{0}\right)}^{3}+H_{4}{\left(\theta -\theta_{0}\right)}^{4}\right]\\ +\underset{\phi }{\sum }\left[V_{1}\left[1-cos\left(\phi -\phi_{1}^{0}\right)\right]+V_{2}\left[1-cos\left(2\phi -\phi_{2}^{0}\right)\right]+V_{3}\left[1-cos\left(3\phi -\phi_{3}^{0}\right)\right]\right]\\ +\underset{\chi }{\sum }K_{\chi }\chi^{2}+\underset{b}{\sum }\underset{b^{\prime }}{\sum }F_{bb^{\prime }}\left(b-b_{0}\right)\left(b^{\prime }-{b^{\prime }}_{0}\right)\\ +\underset{b}{\sum }\underset{\theta }{\sum }F_{b\theta }\left(b-b_{0}\right)\left(\theta -\theta_{0}\right)\\ +\underset{b}{\sum }\underset{\phi }{\sum }\left(b-b_{0}\right)\left[V_{1}cos\phi +V_{2}cos2\phi +V_{3}cos3\phi \right]\\ +\underset{b^{\prime }}{\sum }\underset{\phi }{\sum }\left(b^{\prime }-{b^{\prime }}_{0}\right)\left[V_{1}cos\phi +V_{2}cos2\phi +V_{3}cos3\phi \right]\\ +\underset{\theta }{\sum }\underset{\phi }{\sum }\left(\theta -\theta_{0}\right)\left[V_{1}cos\phi +V_{2}cos2\phi +V_{3}cos3\phi \right]\\ +\underset{\phi }{\sum }\underset{\theta }{\sum }\underset{\theta^{\prime }}{\sum }K_{\phi \theta \theta^{\prime }}cos\phi \left(\theta -\theta_{0}\right)\left(\theta^{\prime }-{\theta^{\prime }}_{0}\right)\\ +\underset{i>j}{\sum }\frac{q_{i}q_{j}}{\varepsilon r_{ij}}+\underset{i>j}{\sum }\left[\frac{A_{ij}}{r_{ij}^{9}}-\frac{B_{ij}}{r_{ij}^{6}}\right] \end{array}$$

where *K_i_*, *H_i_*, *V_i_*, and *F_i_* are constants; *r* is the distance between atoms; *b* is the bond length; *θ* and *φ* are the bond angles of bending and torsion; *q_i_* is the partial charge of a given atom; and *A_i_* and *B_i_* are the van de Waals parameters. The value of these parameters were taken as defined in the COMPASS force field according to the work developed by Sun [18].

**Figure 2:**
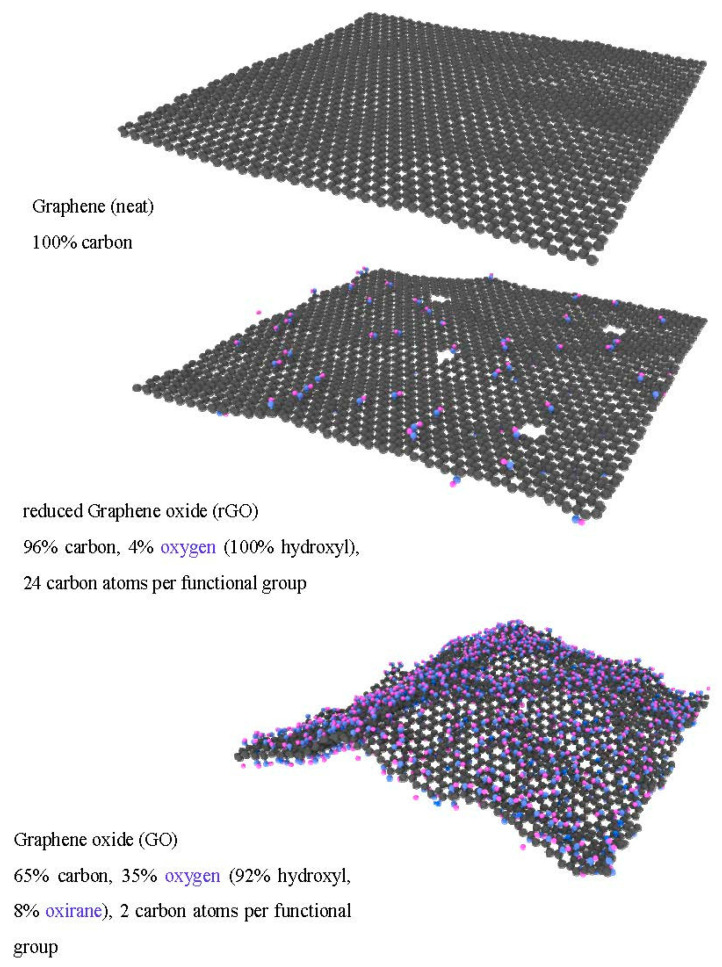
Type of graphene models with details of surface functionalization (percentages are atomic).

**Figure 3:**
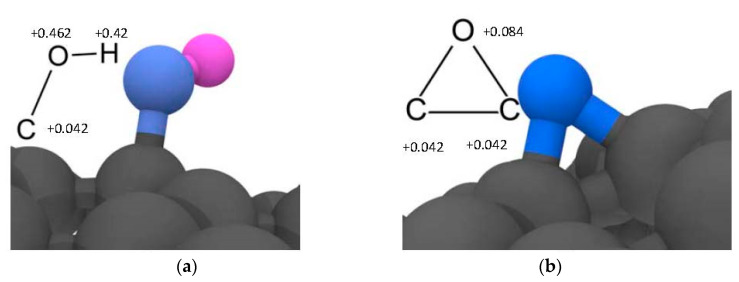
Types of functionalization introduced on the graphene surface: (**a**) hydroxyl group (C-OH) and (**b**) oxirane group (C-O-C). The bonds and the angles interactions between the functional groups and the carbon atoms of the filler were chosen according to the COMPASS force field and the nonbond interactions between the functional groups and polymer molecules.

Three types of graphene fillers (having lateral dimension of 81 × 83 Å, considering periodic boundary conditions in the planar dimensions) were employed to assess the effect of different surface functionalization of the graphene on the interfacial properties (interaction energy and Kapitza resistance) of polymer nanocomposites. In Figure 2, a representative picture of the graphene fillers with details of the type of functionalization and oxidation degree are plotted: neat graphene, graphene oxide (GO) and reduced graphene oxide (rGO). The graphene layer of these fillers was generated using the VMD software [19], whereas the functionalization was introduced considering a random distribution on the filler surface. The bond interactions between filler and functional groups were introduced using LAMMPS [20]. The molecular structures of the two types of functionalization introduced in the graphene layers are shown in Figure 3, i.e., hydroxyl (C–OH) and oxirane (C–O–C) groups. The graphene oxide (GO) contains both types of groups whereas the reduced graphene oxide (rGO) contains only hydroxyl groups. The interaction potential for these graphene fillers was based on a combination of the Tersoff potential [21] for the interaction between the carbon atoms of the graphene layer, and the COMPASS force field [18] for the description of the functional groups (see Figure 3). In detail, the coefficient for bonds and angles between the functional groups and the graphene layer were chosen according to the COMPASS force field (considering the aromatic structure of carbon atoms in graphene).

**Figure 4:**
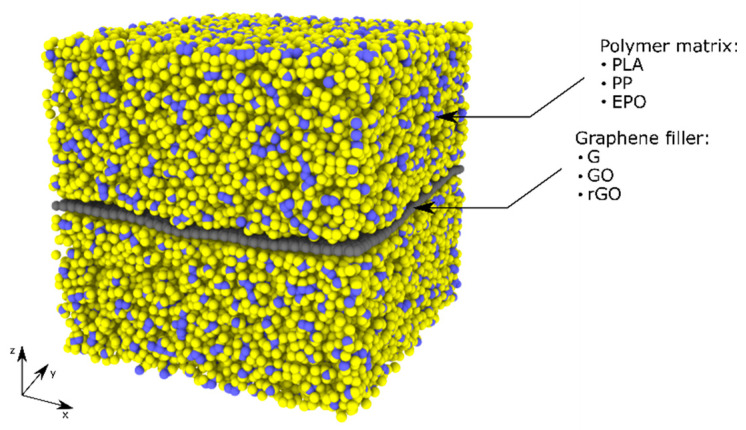
Three-dimensional example of an equilibrated system with PBC and graphene filler that crosses the simulation box (infinite length).

The nanocomposite full atom models (Table 1) were generated by adding the polymer molecules to the cell containing the graphene filler (neat G, GO and rGO). In the case of the thermoplastic polymers, polylactic acid (PLA) and polypropylene (PP), 120 chains with a degree of polymerization of 50 for PLA and 42 chains with a degree of polymerization of 150 for PP (which allow for obtaining representative molecular structures with acceptable computational costs) were added to the cell, whilst for the thermosetting epoxy resin (EPO) composites, DGEBA, DICY and DETA molecules were used in stoichiometric proportion. First, a volume adjustment of the cell was performed; in this step the graphene filler atoms were kept fixed to avoid undesired deformation. Then, the models were time integrated in the isobaric–isotherm ensemble (NPT, with the Nosé–Hoover thermobarostat) at 300 K and 1 atm, allowing all atoms to freely move, until reaching the plateau in density (which typically required between 0.5 and 1 ns). In the case of the epoxy resin, the cross-linking procedure, based on methods described in literature [14,20] and performed through consecutive steps of molecular dynamics simulations, was subsequently applied to obtain the desired cross-linking degree of 70%. At the abovementioned conditions used for the systems stabilizations, the PLA and EPO are in the glassy state, while the polypropylene is in the melting state, as reported in previous studies available in the literature [22,23,24]. The time-step was fixed at 0.5 fs in all simulations and the Verlet algorithm was applied to update the positions of atoms. Periodic boundary conditions (PBC) were applied in all the directions of the simulation cell, which is equivalent to an infinite graphene filler layer (Figure 4). All simulations were performed using the LAMMPS simulation code [20].

### 2.1. Interaction Energy

The interaction energy defines the energy of the system (in terms of the potential energy) implied in the interaction between the polymer matrix and the graphene filler. The interaction energy (Δ*E_i_*) can be calculated considering the energy of the composite and the energy of the isolated components:

(2) $$\Delta E_{i}=E_{tot}-E_{polymer}-E_{filler}$$

where *E_tot_*, *E_polymer_* and *E_filler_* are the potential energies of the composite, polymer and filler, respectively. The potential energy has contributions of bonds, angles, dihedrals, van der Waals and Coulombic interactions (short- and long-range), but not kinetic energy contribution. Indeed, E could represent the total energy or even only a component of the energy (i.e., van der Waals or Coulombic). After the system equilibration in the isothermal–isobaric (NPT) ensemble, the model is finally minimized with the conjugate gradient method using convergence criterions of 0.0 kcal/mol and 1 × 10^−10^ kcal/mol·Å for energy and force. Then, the energy of the composite (*E_tot_*), filler (*E_filler_*) and matrix (*E_matrix_*) were evaluated, pointing out the electrostatic and dispersive contributions. Each partial contribution was calculated, deleting alternatively the filler and the matrix, which allows for extracting the partial potential energy of each phase at its configurational equilibrium with the other phase, neglecting the interaction contribution between them. 

### 2.2. Kapitza Resistance (R_K_)

**Figure 5:**
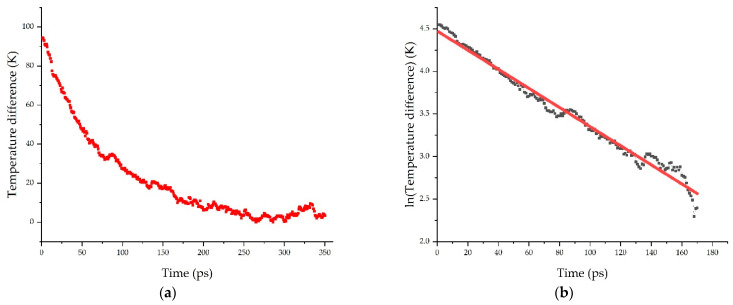
Decay of temperature difference between filler and matrix (**a**), with which the relaxation constant is calculated after linearization (**b**). Curves are obtained from model 2a.

The interfacial thermal resistance, also known as thermal boundary resistance or Kapitza resistance (*R_K_*), is a measure of the resistance of the interface to thermal flow. This property is measured from the atomistic models using a transient approach or nonequilibrium molecular dynamics (NEMD). Initially, the simulation box was entirely equilibrated to the equilibrium temperature of 300 K. Once the whole system (polymer + filler) was equilibrated at this temperature, the filler (graphene) was heated to 400 K (i.e., a temperature delta of 100 K was applied). During the heating process of the graphene filler, the temperature of the polymer was kept constant at 300 K using a second thermostat. Finally, when the filler reached the temperature of 400 K, the thermostats were switched off and the system was allowed to relax in the microcanonical NVE ensemble. During this relaxation process, the temperature of the filler and polymer were monitored, from which the temperature difference over time (*ΔT*(*t*)) was calculated and plotted (some examples are shown in Figure 5). 

The temperature difference (Δ*T*(*t*)) can be fitted using an exponential decay:

(3) $$\Delta T\left(t\right)=\Delta T\left(0\right)\cdot e^{-t/\tau }$$

This expression can be linearized by taking logarithms as follows:

(4) $$ln\Delta T\left(t\right)=ln\Delta T\left(0\right)-\frac{t}{\tau }$$

The Kapitza resistance (*R_K_*) is associated with the relaxation time constant *τ* as stated in the next expression:

(5) $$R_{K}=\frac{A\cdot \tau }{C}$$

where *A* is the filler surface area and *C* the filler heat capacity, which were assumed constant for all the models at ambient conditions [17,25].

### 2.3. Phonon Density of State

The phonon density of states (DOS) of a system describes the proportion of states that are occupied by the system at each energy in the range of modes of vibrations of elastic arrangements of atoms or molecules. The phonons play an important role in the thermal conductivity across the interface between two phases or materials, so the study of this type of vibration mode gives a deep understanding of the influence of atomistic properties on a property such as this [26,27]. The DOS was computed by calculating the Fourier transformation of the atomic velocity autocorrelation functions [28]:

(6) $$\mathrm{DOS}\left(\omega \right)=\int_{0}^{\tau }v\left(t\right)v\left(0\right)\exp\left(-i\omega t\right)dt$$

where *v*(0) and *v*(*t*) are the velocities of each atom at initial time and time *t*, respectively, and *ω* is the frequency. The DOS spectra of graphene fillers and polymers were obtained by monitoring the velocity autocorrelation function of the corresponding atoms. The velocity autocorrelation functions were acquired along 5 ps of trajectory with ten repetitions for statistical average.

## 3. Results and Discussions 

### 3.1. Interaction Energy

**Figure 6:**
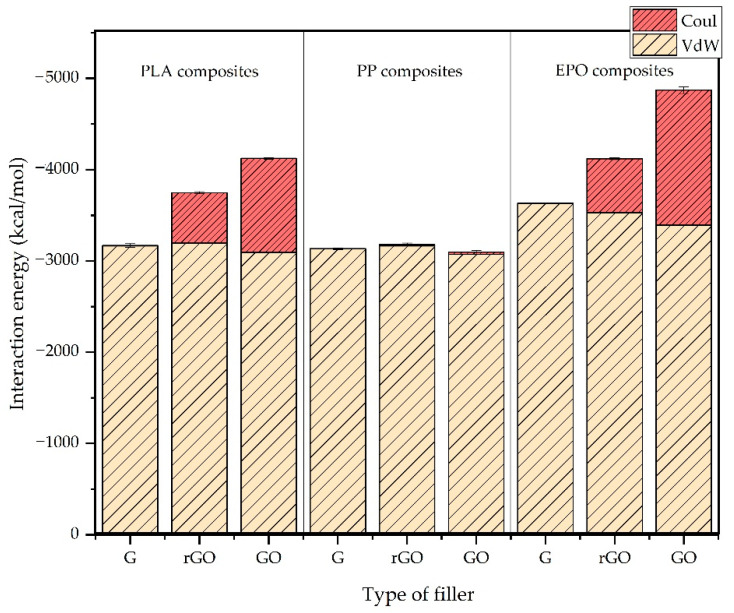
Comparison of interaction energy between the different graphene–polymer composite systems (G: neat graphene; GO: graphene oxide; rGO: reduced graphene oxide).

The interaction energy could be considered as an indicator of the compatibility between two components (in this case between the polymer and the graphene fillers). A negative value of interaction energy means that the interaction produces a release of energy and thus a favorable compatibility between the filler and polymer. The results, which are expressed in terms of average and standard deviation values obtained from three replicas for each system studied, are summarized in Table 2 and plotted in Figure 6 in terms of total (*E*) and van der Waals (*E_VdW_*) or Coulomb (*E_Coul_*) components. In the case of PLA and EPO composite models, the interaction energy between matrix and filler increased in the order G < rGO < GO (Figure 6), or, in other words, when increasing the number of functional groups on the filler surface. This tendency was in good agreement with the work of Sun et al. [15], in which they investigated the interface energy between epoxy resin and graphene with an increasing functionalization degree. On the other hand, PP composites do not present tangible variation in the interactions: G ≈ rGO ≈ GO. This behavior reveals better compatibility of the oxidized fillers (with a particular reference to GO, due to its higher oxidation degree compared to rGO) with PLA and EPO when compared with pristine graphene. In the case of PP, the filler surface oxidation did not affect sensibly the interaction energy, due to the nonpolar nature of this polymer (i.e., no polar groups are present in its composition). The contribution of dispersion forces (van der Waals) and Coulombic interaction to the interaction energy were also assessed from the simulation and, as can be seen in Figure 6, in the case of PP composite interactions, the Coulombic energy represents a negligible contribution to interaction energy. On the other hand, in the case of PLA and EPO, the Coulombic contribution increases sensibly with the filler oxidation degree, inducing an increment on the interaction energy. Then, based on these results, both in the case of PLA and EPO, the interaction energy increases when increasing the level of surface filler oxidation (concentration of oxygen groups), due to the presence of polar groups in the matrixes (hydroxyl (-OH) in the case of PLA and hydroxyl (-OH) and amine (-NH) in the case of EPO), which interact with hydroxyl and oxirane groups of the graphene surface. Finally, the differences in value of interaction energy between the two functionalized fillers depend on the different degree of functionalization. In our case, the GO filler was chosen with a higher concentration of functional groups (35%) than the rGO filler (4%). Based on these results, it was observed that the effect of the oxidation of the graphene filler on the interaction energy with a polymer matrix depended on the polarity of the polymer. In nonpolar polymers (such as PP) the interaction energy was not significatively affected by the oxidation of the graphene, whereas in polar polymers (such as PLA and EPO) the interaction energy was affected by the oxidation of the graphene due to the presence of polar groups in the polymer, which contributed to the Coulombic component.

### 3.2. Kapitza Resistance (R_K_)

**Figure 7:**
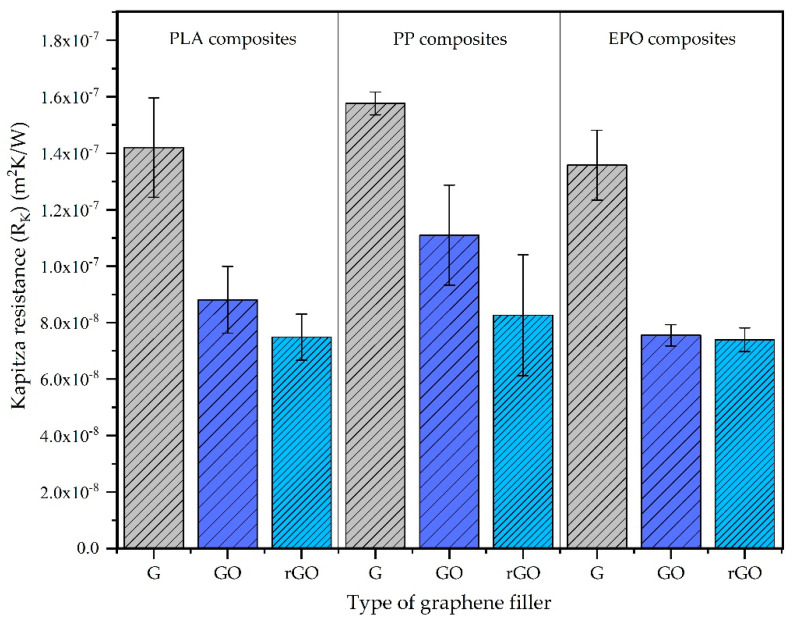
Comparison of Kapitza resistance between the different graphene–polymer composite systems (G: neat graphene; GO: graphene oxide; rGO: reduced graphene oxide).

The results of Kapitza resistance (*R_K_*) (Table 3), which are expressed in terms of average and standard deviation values obtained from the results of three replicas for each studied system, are in the range 0.5–2 × 10^−7^ m^2^K/W, which is in good agreement with the values reported in the literature [11,17]. Considering the effect of the oxidation of the graphene in the interface Kapitza resistance, the same tendency was observed for all the polymers studied (PLA, PP, EPO). Systems reinforced with pristine graphene have the highest values of Kapitza resistance, which decreases when the graphene filler is oxidized (G > GO ≈ rGO) (Figure 7). This behavior indicates that the presence of functional groups in the graphene filler reduces the resistance to thermal heat flow across the interface, which increases the thermal conductivity across the interface. Finally, it is observed that the polarity of the polymer matrix has a less significant influence on the Kapitza resistance, and just the presence of functional groups in the surface of the graphene in form of oxidation influences the thermal conductivity across the interface. Since the heat flux across interfaces is based on a phonon mechanism [29], the DOS spectrum of the studied composite systems is also studied and is further discussed in the next section in order to support these results of Kapitza resistance.

### 3.3. Phonon Density of States (DOS)

**Figure 8:**
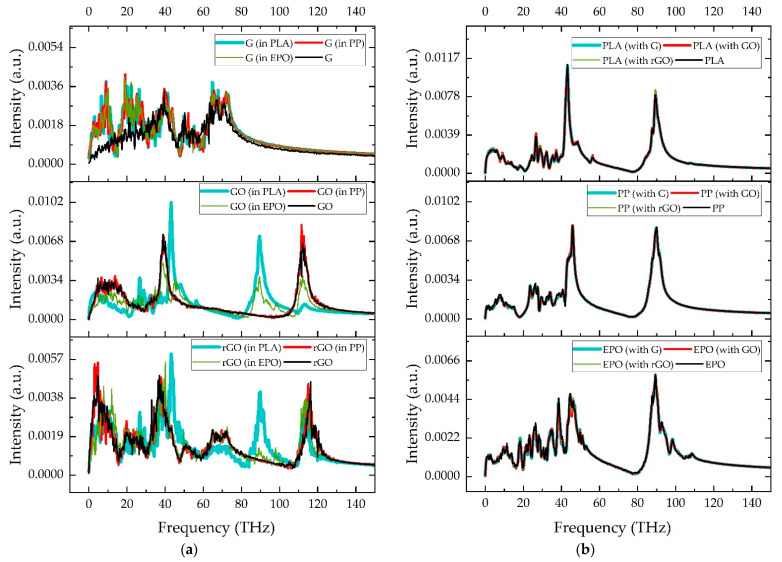
Phonon DOS of (**a**) graphene fillers and (**b**) polymers in composite and neat state (G: neat graphene; GO: graphene oxide; rGO: reduced graphene oxide).

To explain the results of Kapitza resistance, the phonon DOS spectra of graphene fillers and polymers, either in the composite or the neat state, were obtained from the atomic velocity autocorrelation functions after their Fourier transformation (Figure 8). It can be observed that the oxidation clearly alters the phonon vibration modes of the graphene fillers. While the pristine graphene (G) exhibits vibration frequencies below 80 THz, the oxidized graphene fillers exhibit phonon vibration modes at higher frequencies. GO an rGO fillers exhibit an intense phonon vibration mode at near 115 THz, with an additional peak near 90 THz when the graphene was within the PLA or EPO matrices, but not in the case of the PP matrix. These high frequency vibration peaks at 115 THz and 90 THz are both associated with vibrations in which the polar groups (hydroxyl and/or oxirane) are involved, which in the case of the frequency peak at 90 THz presents noncovalent interactions with polar groups of the PLA or EPO matrix that are not present in the PP matrix. The phonon DOS spectra are very similar, in general terms, for the three polymers analyzed. The DOS spectra of the PLA, PP and EPO are dominated by two intense peaks located near 45 THz and 90 THz, with additional vibration peaks at lower frequencies and some extra peaks at higher frequencies in the case of EPO polymer of lower intensity. Whereas the DOS spectra of graphene fillers are affected by the presence of the polymers, as described previously, the DOS spectra of the polymers are practically unaffected by the presence of the graphene fillers. 

Heat carriers of high frequency are important to thermal transport across interfaces [28,30,31]. As previously described, the oxidation of graphene (GO and rGO) provided phonon vibrations at high frequencies that are not present in the pristine graphene (G). These high frequency vibrations enhanced the thermal conductivity across the interface between the polymer and the graphene filler, and thus decreased the associated Kapitza resistance. Moreover, given that the three polymers exhibited similar phonon vibrations according to DOS spectra, the Kapitza resistance of the different composite systems studied is not very dependent on the type of polymer and is mainly affected by the type of graphene filler. In the literature, studies presented by Wang et al. [32] and Zabihi et al. [33] established that when the vibrational frequencies of matrix and polymer were much closer, the phonon transmission probability increases and, thus, the thermal conductivity across the interface increases in the same way. This fact could also explain the results of Kapitza resistance presented in the present study, since the oxidized graphene fillers GO and rGO have phonon vibrations of frequency that are closer to the main polymer phonon vibration frequencies.

## 4. Conclusions

Molecular dynamics (MD) models were developed in order to study the properties of the polymer–graphene fillers interface considering of three different polymer matrices, polylactic acid (PLA), polypropylene (PP) and epoxy resin (EPO) with different graphene derivatives: pristine graphene (G), graphene oxide (GO) and reduced graphene oxide (rGO). From these models, two types of interfacial properties were calculated: the interaction energy and the interfacial thermal resistance (Kapitza resistance). The main conclusions derived from these analyses follow:

- The interaction energy of the three different polymer matrices with the graphene fillers depended on the polarity of the polymer, due to the different contribution of the Coulombic component. Thus, it was observed that the interaction energy increases in the order graphene < rGO < GO for polar polymers such as polylactic acid (PLA) and epoxy resin (EPO), whereas in the case of polypropylene, the functionalization did not affect sensibly its interaction with graphene fillers, due to the negligible Coulombic contribution. These results gave a relevant insight about the matrices–filler compatibility and how it is affected by the different functionalizations considered.
- The Kapitza resistance (RK) exhibited the same behavior/tendency in the three polymers, graphene > rGO ≈ GO. It was observed that the presence of functional groups in the graphene, in the form of oxidation (hydroxyl and oxirane groups) increases the thermal conductivity, which was associated with the appearance of high frequency phonon vibrations in the oxidized graphene fillers, GO and rGO.

## Figures and Tables

**Table 1:** Models studied.

ID Model	Polymer	Filler	N° Atoms	N° Molecules
PLA-G	PLA	G	56,868	120
PLA-GO	PLA	GO	58,596	120
PLA-rGO	PLA	rGO	57,048	120
PP-G	PP	G	59,216	42
PP-GO	PP	GO	61,320	42
PP-rGO	PP	rGO	59,472	42
EPO-G	EPO	G	57,708	1000 + 292 + 164 ^(1)^
EPO-GO	EPO	rGO	59,763	1000 + 292 + 164 ^(1)^
EPO-rGO	EPO	GO	57,888	1000 + 292 + 164 ^(1)^

^(1)^ DGEBA + DICY + DETA number of initial molecules (before cross-linking).

**Table 2:** Interaction energies (in kcal/mol).

ID Model	Polymer	Filler	E_VdW_	E_Coul_	E_total_	Standard Deviation
PLA-G	PLA	G	−3164	−3	−3167	14
PLA-GO	PLA	rGO	−3197	−548	−3745	11
PLA-rGO	PLA	GO	−3091	−1022	−4113	21
PP-G	PP	G	−3132	−0.1	−3132	8
PP-GO	PP	GO	−3073	−24	−3097	17
PP-rGO	PP	rGO	−3151	−14	−3165	21
EPO-G	EPO	G	−3635	1	−3636	4
EPO-GO	EPO	rGO	−3535	−580	−4115	12
EPO-rGO	EPO	GO	−3395	−1461	−4856	34

**Table 3:** Kapitza resistance (*R_K_*) of the systems studied (in m^2^K/W).

ID Model	Polymer	Filler	R_K_	Standard Deviation
PLA-G	PLA	G	1.42 × 10^−7^	0.17 × 10^−7^
PLA-GO	PLA	GO	0.88 × 10^−7^	0.12 × 10^−7^
PLA-rGO	PLA	rGO	0.75 × 10^−7^	0.08 × 10^−7^
PP-G	PP	G	1.58 × 10^−7^	0.04 × 10^−7^
PP-GO	PP	GO	1.01 × 10^−7^	0.27 × 10^−7^
PP-rGO	PP	rGO	0.89 × 10^−7^	0.30 × 10^−7^
EPO-G	EPO	G	1.36 × 10^−7^	0.12 × 10^−7^
EPO-GO	EPO	rGO	0.75 × 10^−7^	0.03 × 10^−7^
EPO-rGO	EPO	GO	0.74 × 10^−7^	0.04 × 10^−7^
